# The different tests for the diagnosis of COVID-19 - A review in Brazil so far

**DOI:** 10.5935/1518-0557.20200046

**Published:** 2020

**Authors:** Ana Flávia Santarine Laureano, Márcia Riboldi

**Affiliations:** 1 Universidade Federal do ABC, Centro de Ciências Naturais e Humanas, São Bernardo do Campo – SP; 2 Igenomix, São Paulo – SP

**Keywords:** Immunochromatography, ELISA, PCR, diagnostic tool, COVID-19

## Abstract

SARS-CoV-2 is a novel virus from the coronavirus family that emerged in the end of December 2019 in Wuhan, China. The virus is now widespread and causing the current pandemic of COVID-19, a highly pathogenic viral pneumonia, commonly presented with fever and cough, which frequently lead to lower respiratory tract disease with poor clinical outcomes associated with older age and underlying health conditions. Supportive care for patients is typically the standard protocol because no specific effective antiviral therapies have been identified so far. The current outbreak is challenging governments and health authorities all over the world. In here we present a comparison among the current diagnostic tools and kits being used to test Brazilian population.

## Background

The coronavirus disease 2019 (COVID-19) epidemic started in December 2019, in Wuhan, Hubei province, in China. It rapidly spread across China and other countries, raising major global concerns (Tang *et al*., 2020). Its etiological agent is the SARS-CoV-2 (Wu *et al*., 2020) also referred to as HCoV-19 (Jiang *et al*., 2020). According to the latest update by the World Health Organization (WHO, 2020a) up to April 28, 2020 there were 2,959,929 confirmed cases with 202,733 deaths in 213 countries, areas or territories so far.

The current COVID-19 outbreak is both similar and different to the prior SARS (2002-2003) and MERS (2012-ongoing) outbreaks. SARS was initiated by zoonotic transmission of a novel coronavirus (likely from bats via palm civets) in markets in Guangdong province, China. MERS was also traced to zoonotic transmission of a novel coronavirus (likely from bats via dromedary camels) in Saudi Arabia. All three viral infections commonly presented with fever and cough, which frequently lead to lower respiratory tract disease with poor clinical outcomes associated with older age and underlying health conditions (Wu & McGoogan, 2020).

The treatment of COVID-19 is supportive. To date, no vaccine, antiviral or other specific treatment is available, however, there are several studies in progress (Wu & McGoogan, 2020). Also, it is not known whether infectiousness starts before onset of symptoms. The incubation period for COVID-19 is about 5-6 days (Li *et al*., 2020a). Combining this time with a similar length serial interval suggests there might be considerable presymptomatic infectiousness (Anderson *et al*., 2020). So far there have been few clinical studies to measure COVID-19 viremia and how it changes over time in individuals (Anderson *et al*., 2020). In one study of 17 patients diagnosed with COVID-19, peak viremia seems to be at the end of the incubation period (Zou *et al*., 2020), pointing to the possibility that viremia might be high enough to trigger transmission for 1-2 days before onset of symptoms.

Diagnostic tests for COVID-19 have stood out in the current coronavirus pandemic as an essential tool for tracking the spread of the disease. The genetic sequence of the 2019 novel coronavirus enabled the rapid development of diagnostics tests specific for SARS-CoV-2 (Wang *et al*., 2020). Since there is a wide range of diagnostic tests commercially available for SARSCoV-2, in this review we present a comparison among of all them and the techniques used to test Brazilian population.

### The Brazilian perspective and diagnostic tests available

**Figure 1:**
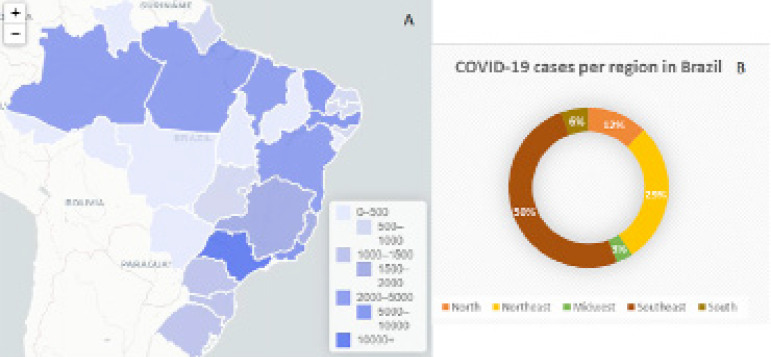
COVID-19 cases in Brazil. Number of cases per state (A) and per region (B) Data collected from State Health Secretaries. Adapted from Brazil, 2020.

 In Brazil, the first case of COVID-19 was confirmed on February 26, 2020 by the Ministry of Health. A 61-year-old man was admitted to a private hospital with a history of travel to Italy, but he was already at home when he presented the symptoms. Since then, on April 25, 2020, 58,509 cases have been confirmed, most of them in the state of São Paulo. Figure 1 illustrates the number of cases per state in Brazil.

 Brazilian Health Regulatory Agency (Anvisa) published the Resolution (RDC 348/2020), which established extraordinary and temporary rules to speed up the evaluation of new products by prioritizing the analysis of test registration requests for detection of the new coronavirus (SARSCoV-2). The idea is not to evaluate and approve products automatically, as sanitary rigor must always exist, but rather to speed up the process. The measure is part of the strategic actions to enable products that can be used to face the COVID-19 pandemic. Anvisa’s role is to promote the protection of the population’s health by executing sanitary control of the production, marketing and use of products and services subject to health regulation, including related environments, processes, ingredients and technologies, as well as the control in ports, airports and borders. On March 19, the Brazilian Health Regulatory Agency approved the first eight rapid tests for the diagnosis of COVID-19. At the date of the review, 39 tests have been approved by Anvisa so far. Of the 39 approved, 21 are rapid tests. There are tests that use blood, serum or plasma and others that need samples of secretions collected from the airways, such as nasopharynx (nose) and oropharynx (throat). Table 1 and 2 summarizes all kits and tests registered in Brazil. Data regarding accuracy have been extracted from manual instructions available at Anvisa website.

**Table 1:** All tests authorized by ANVISA to be used in diagnosing COVID-19 in Brazil so far (NA no available information at 20/04/2020)

Test name	Register number	Process	Manufacturer
**One-Step COVID-2019 test**	**80537410048**	**25351.174464/2020-54**	**Celer Biotecnologia S/A**
**Coronavirus Rapid Test**	**80638720148**	**25351.167156/2020-72**	**Diagnóstica Indústria e Comércio LTDA - ME**
**Coronavirus IgG/IgM (COVID-19)**	**10159820239**	**25351.153719/2020-45**	**Ebram Produtos Laboratoriais Ltda.**
**Medteste Coronavirus 2019-nCov IgG/IgM**	**80560310056**	**25351.189196/2020-75**	**MedLevensohn Comércio e Representações Hospitalares Ltda.**
**Teste rápido em Cassete 2019-nCoV IgG/IgM (blood / serum / plasma)**	**81325990117**	**25351.189190/2020-06**	**QR Consulting Importação e Distribuição de Produtos Médicos Ltda.**
**COVID-19 IgG/IgM ECO Test**	**80954880132**	**5351.148977/2020-18**	**Eco Diagnóstica Ltda.**
**ECO F COVID-19 Ag**	**80954880131**	**25351.162809/2020-27**	**Eco Diagnóstica Ltda.**
**COVID-19 Ag ECO Teste**	**80954880133**	**25351.112132/2020-86**	**Eco Diagnóstica Ltda.**
**Anti COVID-19 IgG/IgM Rapid Test**	**10009010356**	**25351.191493/2020-81**	**Labtest Diagnóstica S/A**
**Detection Kit real time PCR VIASURE SARS-CoV-2**	**10355870373**	**25351.193569/2020-11**	**CerTest Biotec S.L.**
**cobas^®^ SARS-CoV-2**	**10287411491**	**25351.193402/2020-41**	**Roche, Diagnostica**
**Lumiratek COVID-19 (IgG/IgM)**	**81327670112**	**25351.197132/2020-48**	**Lumiradx Healthcare Ltda.**
**Maglumi IgM de 2019-nCoV (CLIA)**	**80102512431**	**25351.206083/2020-41**	**VR Medical Importadora e Distribuidora de produtos Médicos Ltda.**
**Maglumi IgG de 2019-nCoV (CLIA)**	**80102512430**	**25351.206115/2020-17**	**VR Medical Importadora e Distribuidora de produtos Médicos Ltda.**
**Smart Test Covid-19 Vyttra**	**81692610175**	**25351.200980/2020-41**	**Vyttra Diagnósticos Importação e Exportação**

**Table 2:** Performance of diagnostic tests approved by ANVISA in Brazil for COVID-19. (*data extracted from technical instructions from the diagnostic kits available in the register area ate ANVISA website; NA: not available; CI confidence interval).

Test Name	Accuracy data*	Methodology	Sample	Result time
One Step COVID-2019 Test	Sensibility: 86,43% (CI 95%: 82,41-89,58) Specificity: 99,57% (CI 95%: 97,63 - 99,92%)	Immunochromatographic qualitative assay for detection of both IgG and IgM anti-coronavirus	Whole blood, serum or plasma (10 µL)	15min
CORONAVÍRUS RAPID TEST	Sensibility: 86,4% (CI 95%: 82,41% - 89,58%) Specificity: 99,57% (CI 95%: 97,63% - 99,92%)	Immunochromatographic qualitative assay for detection of both IgG and IgM anti-coronavirus	Whole blood, serum or plasma (10 µL)	15min
CORONAVÍRUS IgG/IgM (COVID-19)	IgG Sensibility: >99,9% Specificity: 98,0% Accuracy: 98,6% IgM Sensibility: 85,0% Specificity: 96,0% Accuracy: 92,9%	Immunochromatographic qualitative assay for detection of both IgG and IgM anti-coronavirus	Whole blood, serum or plasma (10 µL)	10-20 min
MedTeste Coronavírus (COVID-19) IgG/IgM (TESTE RÁPIDO)	IgG Sensibility: 97,4% (CI 95%: 86,2% - 99,9%) Specificity: 99,3% (CI 95%: 96,2% - 99,9%) Accuracy: 98,9% (CI 95%: 96,1% - 99,9%) IgM Sensibility: 86,8% (CI 95%: 71,9% - 95,6%) Specificity: 98,6% (CI 95%: 95,0% - 99,8%) **Accuracy: 96,1% (CI 95%: 92,2% - 98,4%)**	**mmunochromatographic qualitative assay for detection of both IgG and IgM**	**Whole blood, serum or plasma (10 µL)**	NA
**Família Teste Rápido em Cassete 2019-nCoV IgG/IgM (sangue total/soro/plasma)**	**IgG** **Sensibility: 100% (CI 95%: 86.0% - 100%)** **Specificity: 98.0% (CI 95%: 89.4% - 99.9%)** **Accuracy: 98.6% (CI 95%: 92.3% - 99.96%)** **IgM** **Sensibility: 85.0% (CI 95%: 62.1% - 96.8%)** **Specificity: 96.0% (CI 95%: 86.3% - 99.5%)** **Accuracy: 92.9% (CI 95%: 84.1% - 97.6%)**	**Immunochromatographic qualitative assay for detection of both IgG and IgM against 2019-nCoV**	**Whole blood, serum or plasma (10µL)**	**10 min**

### Rapid Test or Point of care testing (POCT)

Most rapid tests use colloidal gold particles in a technique known as immunochromatography, also called lateral flow immunoassay, a type of sandwich assay that relies on a pair of antibodies used to recognize two independent epitopes of a protein, and therefore it can achieve high specificity (Zhou *et al*., 2012).

 Lateral flow assays only require the application of a sample (sometimes followed by the application of a buffer solution) and can yield a result within 5-15 minutes (O’Farrell, 2009). This kind of test is being used for pregnancy (Puertas *et al*., 2010), HIV (Granade *et al*., 2010), bacterial infections (Huang, 2007) , drugs of abuse (Gonzalez *et al*., 2011), food contaminants (No *et al*., 2007) and dengue virus (Cuzzubbo *et al*., 2001), and many tests are commercially available (Zhou *et al*., 2012). Lateral flow assays are also being developed for global health applications, where devices that are inexpensive and easy-to-use are required. However, lateral flow assays are generally not quantitative and often only give a yes/no answer (Zhou *et al*., 2012).

 A typical immunochromatographic strip is composed by a sample-loading pad (O’Farrell, 2009), a glass fibre pad with detection antibody (dAb) conjugated to gold nanoparticles (AuNPs) or latex beads (Xu *et al*., 2007), a nitrocellulose or polyvinylidene fluoride membrane with pre-immobilized capture antibody (cAb), a control antibody for test validation (Puertas *et al*., 2010) and an absorbent pad used as capillary pump to draw the sample solution (O'Farrell, 2009).

To perform a lateral flow, assay the sample containing the target analyte (antigen) is loaded on the sample pad and flows through the membrane by capillary effects. The liquid first dissolves the dAb-AuNP conjugates and the antigen binds to the dAb. As the antigen-dAb pair flows through the capture zone, the cAb will capture the labelled antigen. Further downstream, the unbound dAb-AuNP reacts with the control antibody, which binds specifically to dAb irrespective of the antigen. Both the capture and control lines may become visible due to the accumulation of the AuNPs that produce collective plasmonic effects and result in a red colour (Xu *et al*., 2007). The colour on the control line indicates the test is valid, and the colour on the capture line suggests the presence of target analyte in the sample solution (Zhou *et al*., 2012).

**Figure 2:**
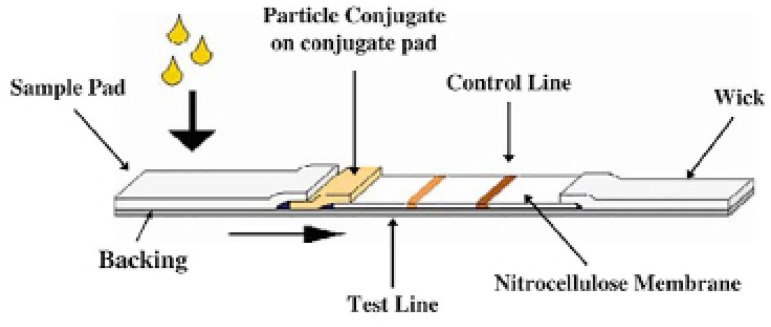
Example of a lateral flow immunochromatographic assay strip (Adapted from O’Farrell, 2009).

For COVID-19 the tests have been developed with AuNPs conjugated with recombinant-anti-COVID-19 antigens. The sample (whole blood, serum or plasma) is added to the pad and, the antibodies against COVID-19 present in the sample, interact with the AuNPs and run through the membrane. When in contact with the test regions these gold-antibodies conjugated are immobilized and a colour line appears on the strip. The presence of the line indicates a positive result. The absence indicates a negative result. There is also a control line, as an indicative that the test is valid. Independent on the result the test is only valid if the control line appears. Figure 2 is an example of how this technology works.

In this context many companies and research groups developed rapid lateral flow immunoassay tests for testing specific antibodies of SARS-CoV-2 in patient blood for being a rapid, simple, highly sensitive diagnosis (Li *et al*., 2020b).

One of the first rapid tests (lateral flow immunoassay) for SARS-CoV-2 IgG and IgM immune responses was developed by professor´s Feng Ye group at the National Clinical Research Centre for Respiratory Disease in Guangzhou, China. The clinical efficiency of the tests was validated by collecting blood samples from 397 PCR confirmed COVID-19 patients and 128 negative patients at 8 different clinical sites. The overall testing sensitivity was 88.66% and specificity was 90.63% (Li *et al*., 2020b). This combined test is being designed and manufactured by Jiangsu Medomics Medical Technologies (Nanjing, China).

From the 39 tests approved 21 are immunochromatographic tests to evaluate the presence of IgG and IgM antibodies against SARS-CoV-2. Sample material for those tests are whole blood, serum or plasma and the time for yielding a result varies from 10-20 minutes. Specificity to IgM antibodies was 94-98% according to the manufacturer while for the IgG was 97-98%. The sensibility for the IgM antibodies was 85-90% and for IgG 95-100%. Three tests - One Step COVID-2019 Test (Celer Biotecnologia S/A), CORONAVÍRUS RAPID TEST (Biocon Diagnósicos) and DPP® COVID-19 IgM/IgG System (Orangelife) had no available data regarding sensibility and specificity for IgG and IgM.

The manufacturers of the tests CORONAVÍRUS IgG/IgM (COVID-19) and 2019-nCoV IgG/IgM Teste Rápido em Cassete reported that hematocrit levels may affect results. The hematocrit level should be between 25% - 65% to yield accurate results. The manufacturer of MedTeste Coronavírus (COVID-19) IgG/IgM informed that results of immunocompromised patients should be interpreted with caution. The manufacturer of the test Anti COVID-19 IgG/IgM Rapid Test declared that no false results were observed in positive sample for the pathogens: influenza A, influenza BRSV, adenovirus, HBsAg syphilis, Helicobacter pylori, HIV and HCV.

In general, this kind of assay shows some advantages such as: it is an established mature technology; relative ease of manufacture since equipment and processes are already developed and available; easily scalable to high volume production, stable, since shelf-lives varies from 12 to 24 months often without refrigeration; ease of use; relatively low cost and short timeline for development and approval (O’Farrell, 2009). However, we have found relatively little current information reporting the diagnostic performance of these POC devices using clinical samples taken from community settings. Relevant data may still be under collection in ongoing studies or may not be published publically.

## ELISA

 The enzyme immunoassays (EIA) and enzyme-linked immunosorbent assays (ELISA) are both widely used as diagnostic tools for the detection and quantification of specific antigens or antibodies in a given sample (Gan & Patel, 2013). Both techniques share similar basic principles and are derived from the radioimmunoassay (RIA). RIA was first described by Berson and Yalow, for which Yalow was awarded the Nobel Prize in 1977, to measure endogenous plasma insulin (Yalow & Berson, 1996). RIA was then developed into a novel technique to detect and measure biological molecules present in exceedingly small quantities, paving the way for more analysis and detection of countless other biological molecules, including hormones, peptides and proteins. Because of the safety concerns regarding its use of radioactivity, RIA assays were modified by replacing the radioisotope with an enzyme, thus creating the modern-day EIA and ELISA (Gan & Patel, 2013).

Both assays use the basic immunology concept of an antigen binding to its specific antibody, which allows detection of small quantities of antigens such as proteins, peptides, hormones or even antibodies in a fluid sample. Those assays utilize enzyme-labelled antigens and antibodies to detect biological molecules; the most used enzymes being alkaline phosphatase (EC 3.1.3.1) and glucose oxidase (E.C. 1.1.3.4). The antigen in fluid phase is immobilized, usually in a 96-well microtiter plate. The antigen can bind to a specific antibody, which is itself subsequently detected by a secondary, enzyme-coupled antibody. A chromogenic substrate for the enzyme yields a visible colour change or fluorescence, indicating then the presence of the antigen. Quantitative or qualitative measures can be assessed based on such colorimetric reading. (Gan & Patel, 2013).

Although ELISA methodology could help track antigen exposure, it has some limitations: the enzyme-mediated colour change will react indefinitely. Over a sufficient long period of time, the colour strength will inaccurately reflect the amount of primary antibody present, yielding false-positive results; to detect a given antibody or antigen, a known reciprocal antigen or antibody must be generated and, nonspecific binding of the antibody or antigen to the plate will lead to a falsely high-positive result (Gan & Patel, 2013).

### Polymerase Chain Reaction

In acute respiratory infection, RT-PCR is routinely used to detect causative viruses from respiratory secretions. PCR is an enzyme-driven process for replicating DNA in vitro. PCR can produce enough amounts of DNA so that pathogens can be detected and identified. Because each pathogen has a unique complement of DNA or RNA, those molecules can function as a molecular fingerprint to help identify what is the organism causing one disease. In this technique a segment of DNA is copied in vitro by using a thermostable DNA polymerase enzyme in the presence of buffer, magnesium, deoxyribonucleoside triphosphates and primers. Oligonucleotide primers complementary to regions on the coding and the noncoding strand of the DNA template are responsible for specificity in the reaction, determining which region of the DNA becomes amplified. As the primers anneal to their complementary regions of DNA, DNA polymerases attach to the primer-template complexes and extend the DNA strands, producing a copy of the DNA. Each copy may then serve as another template for further amplification. Multiple rounds of heating and cooling of the reaction mixture in a thermal cycler produce rounds of melting of the double-stranded DNA, annealing of primer to single-stranded templates, and extension of DNA strands, to produce a logarithmic increase in DNA. In the ideal scenario, the primers chosen in the PCR are specific for a pathogen gene, and hence do not amplify nonspecific targets such as human genes. Theoretically, one could start with a single copy of the target pathogen gene present in the reaction and generate billions of copies of DNA from that gene (Fredricks & Relman, 1999).

There are several approaches for using PCR to detect pathogen DNA, the simplest one being specific PCR where the primers are designed to attach to complementary regions of a DNA target (specific to the pathogen that is being assayed). Broad-range PCR attempt to detect a broader group of organisms by designing primers that are complementary to conserved regions of a particular gene that are shared by a given taxonomic group (Relman, 1998). Another variation is multiplexing, in which multiple specific PCR assays are run simultaneously in the same reaction tube test for multiple different DNA templates. In multiplex PCR several sets of primers are added to the reaction in order to generate several different PCR products. In this case postamplification methods are needed to determine which organism is represented in a positive reaction (Fredricks & Relman, 1999).

PCR is possibly the most quintessential molecular method yet developed. Real-time quantitative PCR (qPCR) revolutionized clinical application of PCR partly because it automated analysis by removing the need for postreaction manipulation (Huggett *et al*., 2015). qPCR is over 20 year old (Higuchi *et al*., 1992), but it has only really been applied clinically in areas which alternatives are not practically possible, such as monitoring treatment in diseases like chronic-phase chronic myeloid leukaemia (Cross *et al*., 2012) or for some key blood borne viruses (Fryer *et al*., 2008).

 Three tests use RT-PCR as the core technology to detect SARS-CoV-2 in samples of the airways of the patients, targeting the conserved genes ORF1ab and N. Probes with fluorescent reporter dye are used to make the detection. Positive results are indicative of the presence of RNA of the virus, but a clinical correlation with patient's history is necessary. Tests are highly sensitive. These data were provided by Secretaria de Ciência, Tecnologia, Inovação e Insumos Estratégicos em Saúde - SCTIE (2020).

### When should we use all the tests?

 On 2 March 2020, WHO released an interim guidance regarding laboratory testing for COVID-19 in suspected human cases recommending that tests should be based on clinical and epidemiological factor and linked to assessment of the likelihood of infection. But is also recommended PCR testing of asymptomatic or mildly symptomatic cases (WHO, 2020b). We should keep in mind that the gold standard for diagnosing COVID-19, as referred by WHO, are nucleic acid amplification tests (NAAT) such as RT-PCR, followed by nucleic acid sequencing when necessary. The viral genes targeted so far include the N, E, S and RdRP genes (WHO, 2020b).

Serological testing can aid investigation of an ongoing outbreak and retrospective assessment of the attack rate or extent of an outbreak. In cases were NAAT assays are negative and there is a strong epidemiological link to COVID-19 infection, paired serum samples (in the acute and convalescent phase) could support diagnosis once validated serology tests are available (WHO, 2020b). It is widely accepted that IgM provides the first line of defence during viral infections, prior to the generation of adaptive, high affinity IgG responses, that are important for long term immunity and immunological memory (Racine & Winslow, 2009). It was reported that after SARS infection, IgM antibody could be detected in patient blood after 3-6 days and IgG after 8 days (Lee *et al*., 2010). Since COVID-19 belongs to the same family of viruses as those that caused MERS and SARS outbreaks it is reasonable to infer that its antibody generation process is similar, and detection of the IgG and IgM antibody against SASR-CoV-2 will be an indicator of infection (Li *et al*., 2020b). Cross reactivity to other coronaviruses can be challenging but commercial and non-commercial serological tests are currently under development (Meyer *et al*., 2014). Some studies with COVID-19 serological data on clinical samples have been published and could help in the development of future tests (Xiao *et al*., 2020).

## CONCLUSIONS

COVID-19 is a novel disease caused by a novel coronavirus (SARS-CoV-2) that emerged in 2019 that is challenging scientists all over the world since its appearance. It is not, however, the first time that coronaviruses are responsible for outbreaks of major importance: SARS (China, 2002-2003) and MERS (Saudi Arabia, 2012-ongoing) were both caused by coronavirus. SARS had an overall case fatality rate (CFR) of 9.6% while MERS presents a CFR of 34.4%. So far, COVID-19 presents a current CFR of 2.6%, however, the total number of COVID-19 cases is likely higher due to inherent difficulties in identifying and counting mild and asymptomatic cases (Wu & McGoogan, 2020) and, it is known that asymptomatic cases act as carriers of SARS-CoV-2. However the mechanism by which asymptomatic carriers could acquire and transmit SARS-CoV-2 still requires further study (Bai *et al*., 2020).

The effort to contain the outbreak is limited by one hard problem: how to differentiate COVID-19 cases from the healthy. For confirmed COVID-19 cases reported the common clinical symptoms include fever, cough, myalgia or fatigue (Huang *et al*., 2020). Yet these symptoms are not unique features of COVID-19 because these symptoms are similar to that of other virus-infected diseases such as influenza (Wang *et al*., 2014).

It is clear the urgent need for rapid, simple to use, sensitive and accurate test to quickly identify infected patients to prevent virus transmission and to assure timely treatment of patients in order to contain this outbreak. We need to acknowledge that all three methodologies exposed here have its advantages and disadvantages and they can and should be combined to address this crisis to map the course of the disease and assure that is not spreading any further.
